# Complete Genome Sequence of Lactobacillus johnsonii Strain Byun-jo-01, Isolated from the Murine Gastrointestinal Tract

**DOI:** 10.1128/MRA.00985-18

**Published:** 2018-10-18

**Authors:** Dongjun Kim, Mun-ju Cho, Seungchan Cho, Yongjun Lee, Sung June Byun, Sukchan Lee

**Affiliations:** a Department of Genetic Engineering, Sungkyunkwan University, Suwon, Republic of Korea; b Animal Biotechnology Division, National Institute of Animal Science, Rural Development Administration, Wanju-Gun, Jeollabuk-do, Republic of Korea; University of Maryland School of Medicine

## Abstract

We report here the complete genome sequence of Lactobacillus johnsonii strain Byun-jo-01, which was isolated from the murine gastrointestinal tract. The genome was determined using both PacBio and Illumina sequencing. L. johnsonii strain Byun-jo-01 contains a single circular chromosome of 1,959,519 bp, and its GC content is 34.7%.

## Abstract (precis)

We report here the complete genome sequence of Lactobacillus johnsonii strain Byun-jo-01, which was isolated from the murine gastrointestinal tract. The genome was determined using both PacBio and Illumina sequencing.

## ANNOUNCEMENT

Various microbial species live in the murine gastrointestinal tract, and their interactions can have a beneficial effect on the host (1). Among these intestinal microbes, Lactobacillus species in the small intestine are particularly beneficial. More than 100 species belonging to the genus Lactobacillus have been identified (2, 3), and their genome sequences are available because of modern next-generation sequencing (NGS) techniques (4–8). There have been attempts to express antibodies or other proteins on the surface of Lactobacillus species for use as vaccines (9). Similarly, mixing Lactobacillus species with prebiotics or other proteins could maximize their probiotic effect on the host. In a previous study, L. paracasei strain ATCC 334 expressing 3D8 scFv (a nucleic acid hydrolyzing miniantibodies) showed antiviral effects against murine norovirus, which could be used as a potential antiviral agent (10). The purpose of this study was to select potential probiotics from the murine small intestine to be used as antiviral food additives.

Here, we report the complete genome of L. johnsonii strain Byun-jo-01, isolated from the jejunum of a 6-week-old female ICR mouse (DBL, Republic of Korea). All animal procedures performed in this study were reviewed, approved, and supervised by the Institutional Animal Care and Use Committee (IACUC) of Konkuk University (KU16080). All experimental procedures performed were in accordance with the guidelines of the Institute of Laboratory Animal Resources (ILAR). The jejunum, a part of the small intestine, was harvested, and tissue samples were homogenized using 1.6-mm stainless steel beads. Homogenized tissue samples were serially diluted 10-fold in phosphate-buffered saline and spread onto de Man-Rogosa-Sharpe (MRS) agar, which is able to select Lactobacillus species, and then cultured at 37°C for 2 days in an anaerobic environment. Among the many colonies, only one colony randomly was picked from the MRS agar and incubated in MRS medium for 2 days. These bacterial isolates were selected based on their rod-shaped morphology using microscopy. The sequences of 16S rRNA and DNA gyrase subunit B were 99% similar to those of most other L. johnsonii strains, including L. johnsonii strain NCC 533 (as reported in the NCBI database).

Genomic DNA was prepared using a mixture of lysozyme and mutanolysin in Tris-EDTA (TE) buffer in combination with the G-spin genomic DNA extraction kit (iNtRON, Republic of Korea) (11). Extracted DNA was sequenced by two methods to assemble the complete chromosome sequence. The genome of L. johnsonii strain Byun-jo-01 was sequenced at Macrogen (Seoul, Republic of Korea) using both the HiSeq 2000 (2 × 100-bp paired-end sequencing; Illumina, Inc., USA) and RS II (Pacific Biosciences, USA) platforms. Single-molecule real-time (SMRT) cell sequencing with 20-kb inserts generated 1,847,047,342 bp (182,640 reads, about 635× coverage). A total of 182,640 reads were assembled into a single contig by the Hierarchical Genome Assembly Process (HGAP3) using SMRT Analysis software version 2.3.0 (12). Additionally, paired-end libraries with insert sizes of 300 bp were prepared using the NEBNext Ultra DNA library prep kit for Illumina sequencing with the HiSeq 2000 platform, which produced 81,648,222 reads (100-bp read length). To improve draft genome assemblies, hybrid assembly was conducted using Pilon version 1.21 with short reads generated from Illumina sequencing. By these methods, the complete genome size of L. johnsonii strain Byun-jo-01 was determined to be 1,959,519 bp, and its GC content is 34.7%. Putative coding sequences were annotated with Prodigal version 2.6 software (13). The tRNA and rRNA genes were predicted using ARAGON version 1.2 and RNAmmer version 1.2 software, respectively (14). Default parameters were used for all software during the assembly and annotation process. The genome of L. johnsonii strain Byun-jo-01 comprises 77 tRNAs and 21 rRNAs. A total of 1,781 protein-coding sequences were discovered.

### Data availability.

The chromosomal sequence of L. johnsonii strain Byun-jo-01 has been deposited in GenBank under the accession number CP029614.
